# Quantitative methods in microscopy to assess pollen viability in different plant taxa

**DOI:** 10.1007/s00497-020-00398-6

**Published:** 2020-10-29

**Authors:** Lorenzo Ascari, Cristina Novara, Virginia Dusio, Ludovica Oddi, Consolata Siniscalco

**Affiliations:** Department of Life Sciences and Systems Biology, University of Turin, Viale Mattioli, 25, 10125 Turin, Italy

**Keywords:** Clustering, Random forest, Image analysis, Fluorescein diacetate (FDA), Propidium iodide (PI)

## Abstract

High-quality pollen is a prerequisite for plant reproductive success. Pollen viability and sterility can be routinely assessed using common stains and manual microscope examination, but with low overall statistical power. Current automated methods are primarily directed towards the analysis of pollen sterility, and high throughput solutions for both pollen viability and sterility evaluation are needed that will be consistent with emerging biotechnological strategies for crop improvement. Our goal is to refine established labelling procedures for pollen, based on the combination of fluorescein (FDA) and propidium iodide (PI), and to develop automated solutions for accurately assessing pollen grain images and classifying them for quality. We used open-source software programs (CellProfiler, CellProfiler Analyst, Fiji and R) for analysis of images collected from 10 pollen taxa labelled using FDA/PI. After correcting for image background noise, pollen grain images were examined for quality employing thresholding and segmentation. Supervised and unsupervised classification of per-object features was employed for the identification of viable, dead and sterile pollen. The combination of FDA and PI dyes was able to differentiate between viable, dead and sterile pollen in all the analysed taxa. Automated image analysis and classification significantly increased the statistical power of the pollen viability assay, identifying more than 75,000 pollen grains with high accuracy (*R*^*2*^ = 0.99) when compared to classical manual counting. Overall, we provide a comprehensive set of methodologies as baseline for the automated assessment of pollen viability using fluorescence microscopy, which can be combined with manual and mechanized imaging systems in fundamental and applied research on plant biology. We also supply the complete set of pollen images (the FDA/PI pollen dataset) to the scientific community for future research.

**Electronic supplementary material:**

The online version of this article (10.1007/s00497-020-00398-6) contains supplementary material, which is available to authorized users.

## Introduction

To complete the reproductive phase, angiosperms, conifers and gnetophytes develop pollen grains capable of germinating on a receptive gynoecium and develop a specialized tube as a conduit to the female ovule through one of the fastest-growing cellular structures in the natural world (Bedinger 1992) and fertilize the egg cell. The competence to accomplish all the steps required for successful seed-set was defined together as pollen performance traits (Williams and Mazer 2016). Among the parameters of pollen performance, pollen viability is one of the fastest and easiest to assess, because it does not require waiting for seeds to set or pollen tubes to emerge. The importance of pollen viability itself for estimating success in plant reproduction has been studied in detail through estimates of pollen viability and was found to be strongly related to pre-zygotic success (Alonso et al. 2013; Arceo-Gómez and Ashman 2014). For example, a higher quality cross pollen was able to improve both fruit set and fruit nutritional properties in almonds (*Prunus dulcis*) (Brittain et al. 2014; Klein et al. 2015). Moreover, studies on *Mimulus guttatus* showed how pollinators preferred outbred plants with a higher pollen viability compared to inbred lines with lower quality pollen (Carr and Dudash, 1997; Carr et al. 2014; Yeamans et al. 2014). Both genetic and environmental factors can shape pollen viability. Inbreeding depression and natural hybridizations were also shown to negatively influence pollen viability (Bureš et al. 2010; Eppley and Pannell 2009), whereas environmental stress can impact male gametogenesis at different developmental stages (De Storme and Geelen 2014). Water balance, in particular, plays an important role, with dehydration being associated with sharp viability reduction in some sensitive pollen species (Chichiriccò 2000; Fonseca and Westgate 2005; Nepi et al. 2010). On the contrary, tightly controlled hydration is required for full metabolic activation and pollen tube emergence (Edlund et al. 2004). Various methods were developed to assess pollen viability, based on pollen sizes (Kelly et al. 2002) or on dielectric properties of cell membranes (Heidmann et al. 2016). Despite the introduction of new methodologies, most of the scientific community still relies on classical techniques that only require simple staining procedures (Shivanna and Tandon 2014) and a microscope for manual counting. Consequently, due to the considerable efforts required, in general, only a few hundreds of grains per replicate are counted at best. Image analysis software was used for the automation of the tests based on Alexander’s and aniline blue staining (Mudd and Arathi 2012; Tello et al. 2018). Nevertheless, those stains are suited for identifying aborted or sterile grains and not for studying the state of well-developed pollen (Alexander, 1969; Khatun and Flowers 1995). The double labelling technique that employs fluorescein diacetate (FDA) and propidium iodide (PI) has been used effectively to label dead and viable plant protoplasts (Huang et al. 1986) and sperm cells (Zhang et al. 1992). Greissl (1989) was the first to suggest the use of FDA and PI to evaluate pollen quality. Being able to label both dead and viable pollen, this combination usually improves classification accuracy at low viability levels in comparison with FDA alone (Aronne et al. 2001). Fluorescein diacetate accumulates inside the cytoplasm of viable pollen grains with intact plasma membranes, upon hydrolysis to fluorescein by intracellular esterases through the fluorochromatic reaction (FCR) (Heslop-Harrison and Heslop-Harrison 1970), whereas propidium iodide labels pollen wall following its higher affinity for pectins (Rounds et al. 2011). The same technique was also used for the assessment of pollen sterility levels (Colombo et al. 2017). In our study, different approaches for the quantitative evaluation of pollen quality using the FDA/PI labelling were tested. The purpose of this work was (1) to assess pollen viability and sterility in various taxa by manually tagging viable, dead and sterile grains based on the FDA/PI combination, (2) to develop two alternative automated approaches for image processing and pollen counting using free open-source software, (3) to employ machine-learning techniques for the supervised and unsupervised classification of counted pollen into relevant populations, (4) to compare the results of the automated approaches to the classical manual procedure and (5) to generate a publicly available dataset of single pollen images labelled with FDA/PI for future research in computer vision. In general, our work aims to supply plant biologists with automated solutions as a reliable alternative to manual counting allowing a statistically sound investigation of plant male reproductive performance.

## Materials and methods

### Plant material and sample preparation

Mature anthers from *Solanum lycopersicum*, *Clivia miniata *(20030012), *Malus domestica *(19980025), *Magnolia stellata*, *Actinidia deliciosa *(20020032), *Olea europea* and fresh pollen from male flowers of *Quercus suber* were collected at the Botanical Garden of Turin (Italy). Fresh pollen was also collected from plants of *Corylus avellana* ‘Tonda di Giffoni’ (TG) grown in a private orchard located in Cunico (Piedmont, Italy) and from two distinct wild hazelnuts (*wt*) growing in the woods surrounding the same orchard. A common storage protocol was employed for both anthers and dehisced pollen: samples were dehydrated overnight in sealed boxes using silica gel and stored at − 18 °C. The analysis of samples occurred within three months from collection. Before the analysis, samples were thawed out and rehydrated for one hour inside Petri dishes with wet blotting paper at the bottom (Shivanna and Rangaswamy 1992). To ensure pollen release and collection avoiding damage, anthers were either mildly washed by hand or vortexed for very short times in Brewbaker and Kwack (BK) medium (Brewbaker and Kwack 1963) and filtered using 200 µm strains. Dehisced pollen was analysed immediately after rehydration without any additional step.

### Labelling and imaging

Samples were labelled employing a revised FDA/PI labelling technique optimized for pollen grains. A small amount of rehydrated pollen was gently mixed in BK medium with the addition of PI (1 mg/ml in phosphate buffer saline) for a final concentration of 20 µg/ml and FDA (4 mg/ml in acetone) for a final concentration of 8 µg/ml. After 5 min of incubation in the dark, pollen samples were centrifuged at 7000 rpm for two minutes and the supernatant was removed and replaced with clean BK medium. The washing procedure was repeated two times to remove label leftovers. Finally, pollen was resuspended in a small volume of BK medium and laid on microscope slides for imaging. All solutions were prepared immediately before use, and exposition to light was avoided as much as possible during washing, imaging and transitional steps. For acquiring the images, a Nikon Eclipse E400 epi-fluorescent microscope with B-2A filters and a Nikon Digital Sight DS-U1 camera system were used. Images were taken at a resolution of 640 × 480 pixels and 100 × magnification, from three microscope slides for each taxon and pooled together for the following analysis. A minimum of 90 and a maximum of 151 per-taxon images were collected.

### Manual counting (MC) of pollen grains

**Fig. 1:**
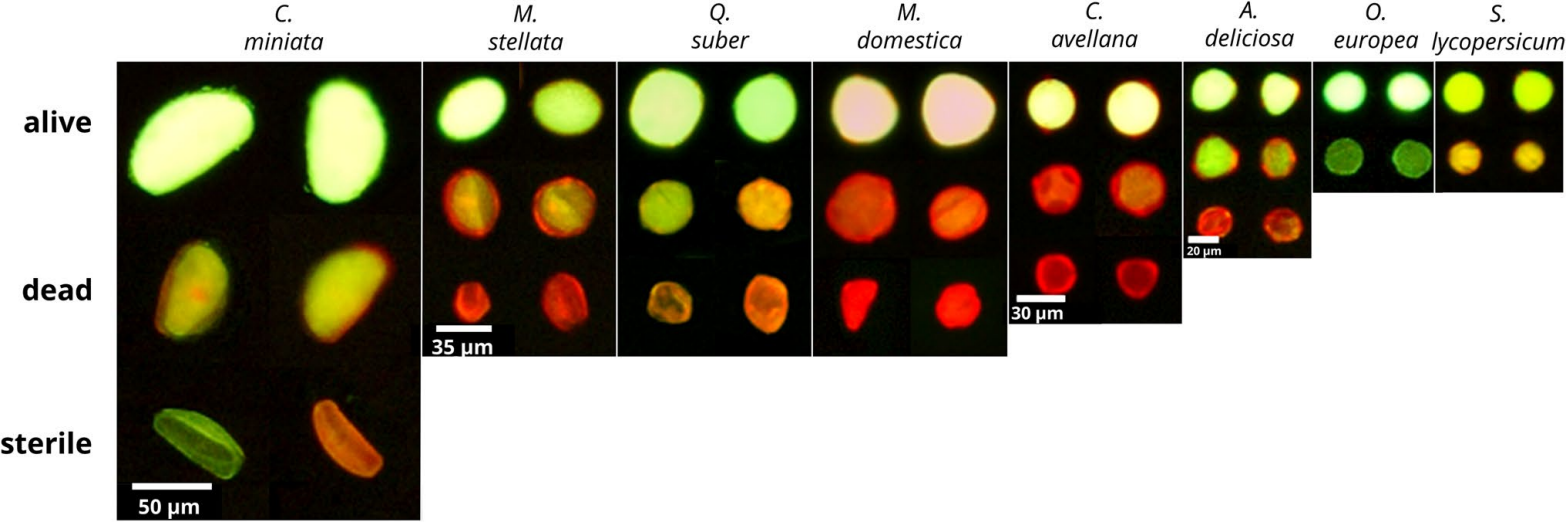
Collection of images for each pollen taxon included in the study. Two examples of viable, dead and sterile pollen grains can be found in the first, second and third rows, respectively. In *O. europea* and *S. lycopersicum*, sterile grains were not present. Image background was corrected using the CellProfiler pipeline

Around 1500 pollen grains for each taxon were manually tagged on random images using the “Cell Counter” plugin included in the Fiji platform. Pollen with bright green or yellow fluorescence was classified as viable, while pollen with dim fluorescence was labelled as dead. Smaller, collapsed pollen mostly unable to retain fluorescein was considered as sterile (Fig. 1). The same evaluation scheme was used for the supervised and unsupervised classification procedure.

### Automated image analysis and classification of pollen viability

**Fig. 2:**
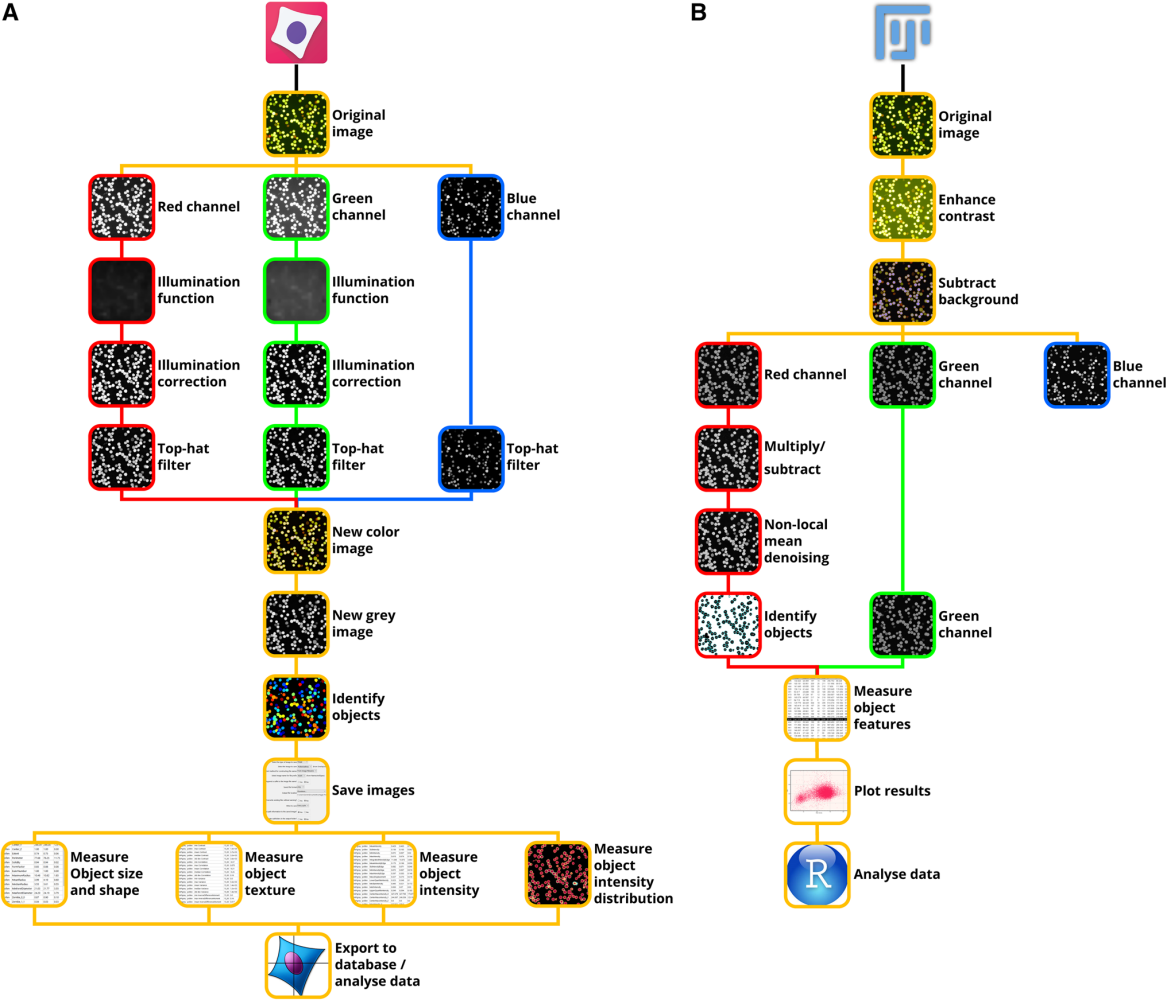
Image processing, object identification and feature extraction procedures developed using **a** CellProfiler and **b** Fiji software. See main text for detailed description

For the automatic counting of pollen grains in the acquired images, two different approaches were developed using free open-source programs, CellProfiler (McQuin et al. 2018) and Fiji (Schindelin et al. 2009). Figure 2 shows the workflow followed for the analysis of images with the two software programs.

#### CellProfiler analysis

The first strategy (Fig. 2a) utilized CellProfiler modules to split the parental RGB images into the three channels. For each image set, a Gaussian filter calculated the illumination functions for the red and green channels. The calculated illuminations were subtracted to the original grey images to reduce background unevenness. Additionally, a top-hat filter reduced extra background signal from the red, green and blue channels. New colour and grey images were obtained by merging the corrected RGB channels. The newly generated grey images were thresholded in three classes by the Otsu algorithm, and clumped objects were separated following shape indentations. Objects either too small or too large and touching image borders were discarded. New colour images and object outlines were saved for further analysis. For each taxon, 374 features related to object position, size, shape, texture, colour intensity and intensity distribution were calculated and saved to a local SQLite database for data analysis. Intensity parameters were measured on the reconstructed grey images. A sample pipeline is provided for reproducibility in file S1.

#### Fiji analysis

A macro was programmed with the Fiji platform for the second image analysis method (Fig. 2b). A sample file with the complete procedure can be found in file S2. The original pollen images belonging to the different plant taxa were pre-processed enhancing the contrast by histogram equalization. Image background was corrected via the rolling ball algorithm (Sternberg 1983) applied on the whole image stack. Subsequently, images were split into the three RGB channels and the blue channel was discarded. The signal in the red channel was optionally enhanced through multiplication and subtraction operations. In order to further reduce background signal, denoising was applied by way of non-local-means (Buades et al. 2011). Different thresholding methods were applied depending on the pollen taxon for image segmentation and object recognition. Pollen grains were separated using an adjustable watershed algorithm, and pollen touching the image borders was discarded. Using “particle analyser” plugin, 33 features related to object position, size, shape and labelling intensity were calculated. The information about labelling intensity was measured on the green channel images. A dimensional range was set in order to exclude overly large (i.e. pollen clumps) and small objects (i.e. debris). A final plot was shown after each analysis in order to assess the results before saving them to a local csv database.

#### Supervised classification

For the supervised classification (SC) of pollen viability, per-object features extracted with CellProfiler software were analysed with the companion CellProfiler Analyst, a free, user-friendly tool for supervised classification that requires no programming skills (Dao et al. 2016). Data were firstly explored using density plots and manual gating (Fig. S1a**)** for the identification of relevant populations (i.e. sterile, viable, dead pollen and debris). Features related to objects locations were removed from the analysis as not useful for the classification process. Moreover, using the “classifier” tool (Fig. S1b), image thumbnails of pollen grains and debris were randomly fetched and conferred to user-defined classes (viable, dead, sterile and debris) to build a balanced annotated dataset that was used for model training, with 40 objects for each class. This was chosen as a minimum valid number considering the complexity of the classification and the number of classes and to make the training operation less time-consuming. Training was carried out on the annotated dataset using a random forest algorithm. Classification performance on the training set was monitored by computing accuracy and Cohen’s Kappa statistics, while partial accuracy on the remaining data was verified by scoring sample images (Fig. S1d). Finally, the whole experiment was scored and per-class counts were computed.

#### Feature selection and unsupervised clustering

**Fig. 3:**
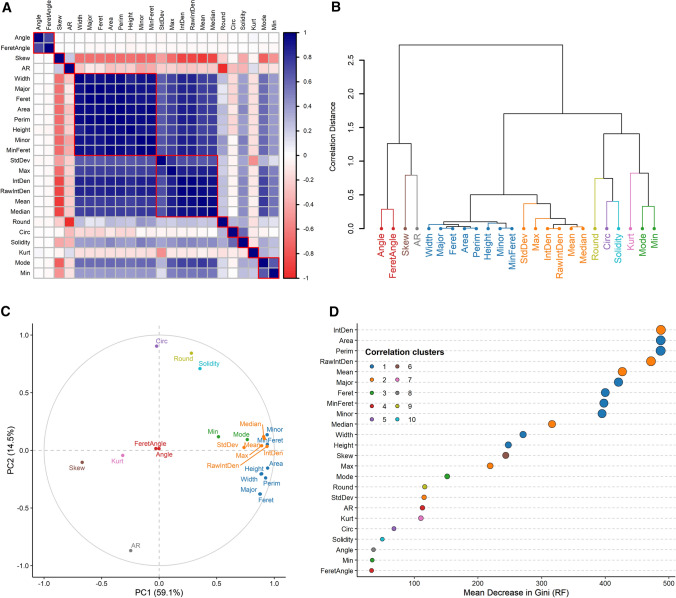
Example of analysis in the R environment of pollen features gathered by the Fiji workflow on *C.avellana* (TG) images. **a**, **b** Spearman correlation distances between pollen features grouped through hierarchical clustering. **c** Contribution of pollen features to the first two principal component of the variance. **d** Gini importance for pollen features computed by an unsupervised random forest. Colours in **b–d** correspond to clusters of correlation distances. For complete feature names and description, see Fiji manual

The R environment (R Core Team 2019) was used for the unsupervised clustering (UC) of viability data collected by the Fiji workflow (code available in additional file S3). Pollen features were scaled, and the characteristics related to pollen positions on images were removed. To select the three most important features for the automated identification of viability clusters, a multi-step procedure was applied: (1) after checking for data normality, Spearman correlation distances between features were computed and grouped by hierarchical clustering (Fig. 3a, b), (2) principal component analysis (PCA) was applied to assess feature contribution to the first two principal components (Fig. 3c), (3) using package “randomForest” (Wiener 2002), a random forest was run in unsupervised mode for the calculation of the mean decrease in Gini importance (GI) as a measure of feature relevance (Fig. 3d). Furthermore, Manhattan distances among the three selected variables were used to determine the best number of clusters through the consensus between 26 indices calculated by the package “NbClust” using the k-means algorithm (Charrad et al. 2014). By using the identified number of clusters, hierarchical clustering on Manhattan distances between pollen features was utilized to identify the populations of viable, dead and sterile pollen grains. The same procedure was repeated for each of the analysed pollen taxa.

### Method comparison

To assess the ability of the Fiji macro and CellProfiler pipeline to correctly identify pollen grains on images, linear regressions (*R*^*2*^) and Bland–Altman analyses were performed between per-image total pollen counts measured with manual and automated procedures. Bland–Altman plots were also used for measuring classification accuracy of viable, dead and sterile pollen for UC and SC methods compared against the MC results. The presence of proportional bias (when the values of the differences change in proportion to averages) in Bland–Altman plots was estimated through linear regression and *t* test for slope significance (*P* ≤ 0.05) (Altman and Bland 1999). Heteroscedasticity (when the variance of the differences changes in proportion to averages) was accounted for the implementation of 95% V-shaped confidence limits (Ludbrook 2010). For every class of dead, viable and sterile pollen, the nonparametric Kruskal–Wallis test and the post hoc Wilcoxon signed-rank test (*P* ≤ 0.05) evaluated the presence of significant differences between mean percentages computed by the three methods within each pollen taxon. The entire analysis was performed in the R environment (R Core Team 2019).

### Generating the pollen image dataset

Individual pollen images were generated using CellProfiler software by shrinking objects’ masks to their centers, expanding them by a defined number of pixels and applying the new masks to the corrected images. One of the hazelnut samples was considered redundant and excluded from the pollen dataset generation. Finally, the image dataset was cleaned from over segmented pollen grains and unwanted debris.

## Results

### Measuring pollen identification accuracy across automated counting methods

**Fig. 4:**
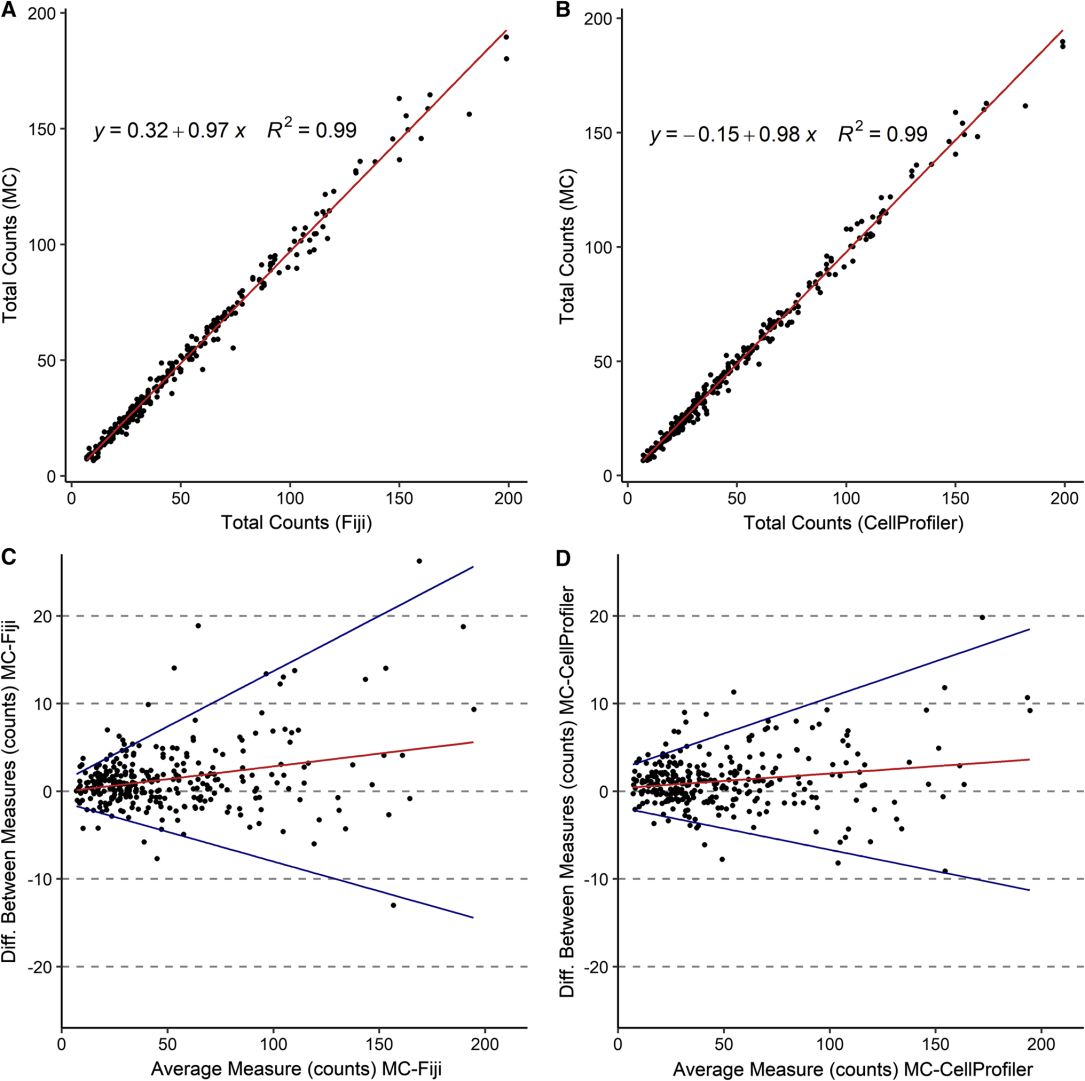
Linear regression (*R*^*2*^) and Bland–Altman analyses to assess identification accuracy of pollen grains on images between **a**, **c** Fiji macro and manual counting (MC) and **b**, **d** CellProfiler pipeline and manual counting (MC)

Overall, 15,340 pollen grains were counted on 304 images using the manual method. 76,120 and 75,329 pollen grains were counted on 1187 images by the CellProfiler pipeline and the Fiji macro, respectively (Table S1). Linear regression computed on per-image total pollen counts gave *R*^*2*^ values close to one for both Fiji macro and CellProfiler pipeline compared against MC (*R*^*2*^ = 0.99, Fig. 4a, b), underlining the close relationship between automated and manual methods. The Bland–Altman analysis (Fig. 4c, d) showed that both methods progressively underestimated pollen counts, while the average number of pollen grains on images increased (*y* = 0.02 + 0.3, *P* ≤ 0.0001 * y*= 0.03 − 0.1, *P* ≤ 0.0001, respectively, for CellProfiler and Fiji procedures), albeit this trend was more pronounced for the Fiji macro. Moreover, the variance of differences between automated and manual values also increased at higher average counts, especially in the Fiji procedure. Deviations in counted pollen per taxon between manual and automated methods ranged from − 420 to + 15 for the Fiji macro and from − 358 to 94 for the CellProfiler pipeline (Table S1).

### Supervised classification performance

Supervised classification performance using the random forest classifier considering all the plant taxa analysed in this study was characterized by an overall accuracy of 74% (95%, CI 0.7142–0.7646) and a Kappa Cohen’s statistic of 65% (Fig. S1c).

### Characterization of pollen features collected with the Fiji macro

**Fig. 5:**
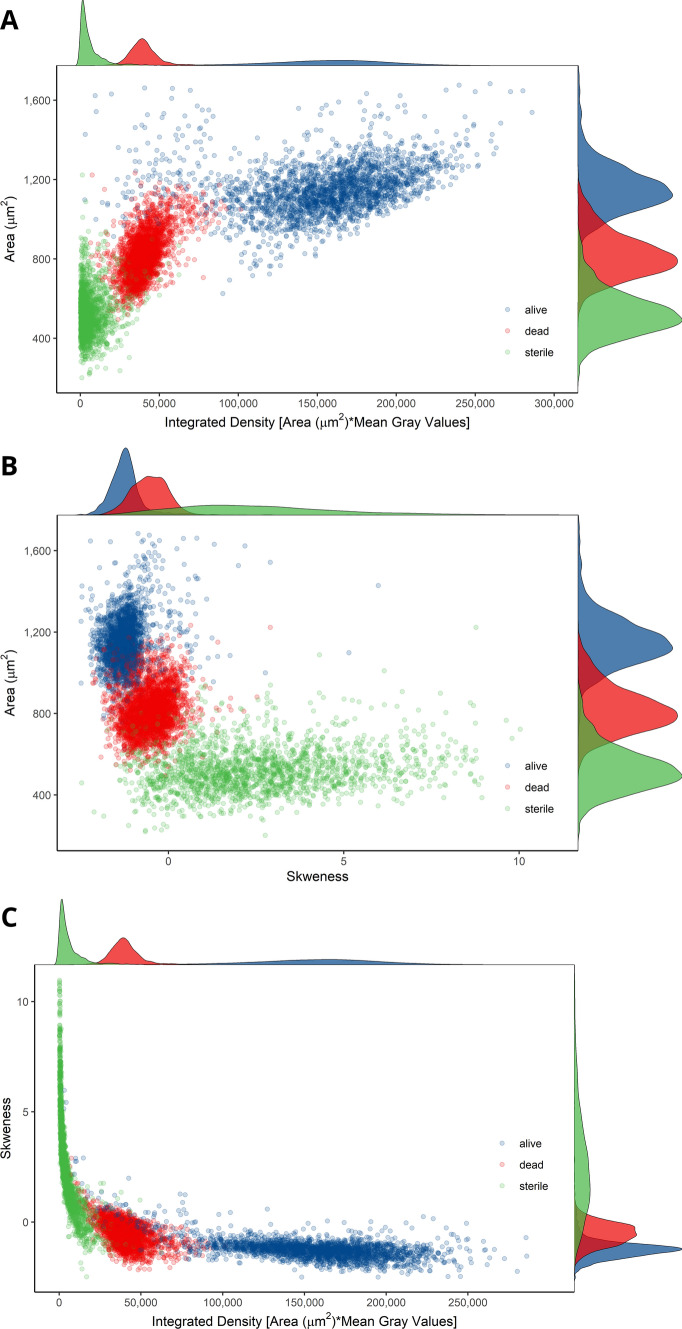
Populations of viable, dead and sterile pollen grains of *C. avellana* (TG) identified by the unsupervised clustering of pollen features measured by the Fiji macro. Plotted are the three main variables resulting from the features selection procedure. **a** Pollen grain area (µm^2^) vs integrated density [area (µm^2^) × mean grey values] **b** Area (µm^2^) versus skewness, **c** skewness versus integrated density [area (µm^2^) × mean grey values]

The feature selection procedure carried out on the data obtained by the Fiji macro from the images of *C. avellana* TG pollen identified two main groups of features, whose reciprocal similarity was stressed by strong positive correlations (Fig. 2a). The first group was related to pollen colour intensity and the second was associated with pollen dimension (Fig. 2a). Within these groups, integrated density [area (µm^2^) × mean grey values] and average area (µm^2^) were ranked as the two most important features by the GI computation (Fig. 2d). Skewness, a coefficient describing asymmetries of pixel value distributions in the identified objects, was negatively correlated to the first two main groups of features (Fig. 2a) and to the first principal component of the variance (− 0.67, *P* ≤ 0.0001, Fig. 2d). Moreover, random forest ranked skewness in the third place in terms of importance, after intensity and dimension groups (Fig. 2d). Viable and dead pollen grains were mainly separated along the dimensional and colour intensity gradient (Fig. 5a), whereas skewness was determinant for classifying the population of sterile pollen (Fig. 5b, c). Integrated density, area and skewness were also necessary for the estimation of the best number of clusters. Similar results were obtained by the analysis on all the remaining pollen taxa (data not shown). Scatterplots and results of the hierarchical clustering of Manhattan distances among pollen features for all the analysed taxa are found in figure S2.

### Measuring classification performance of pollen viability and sterility across automated counting methods

**Fig. 6:**
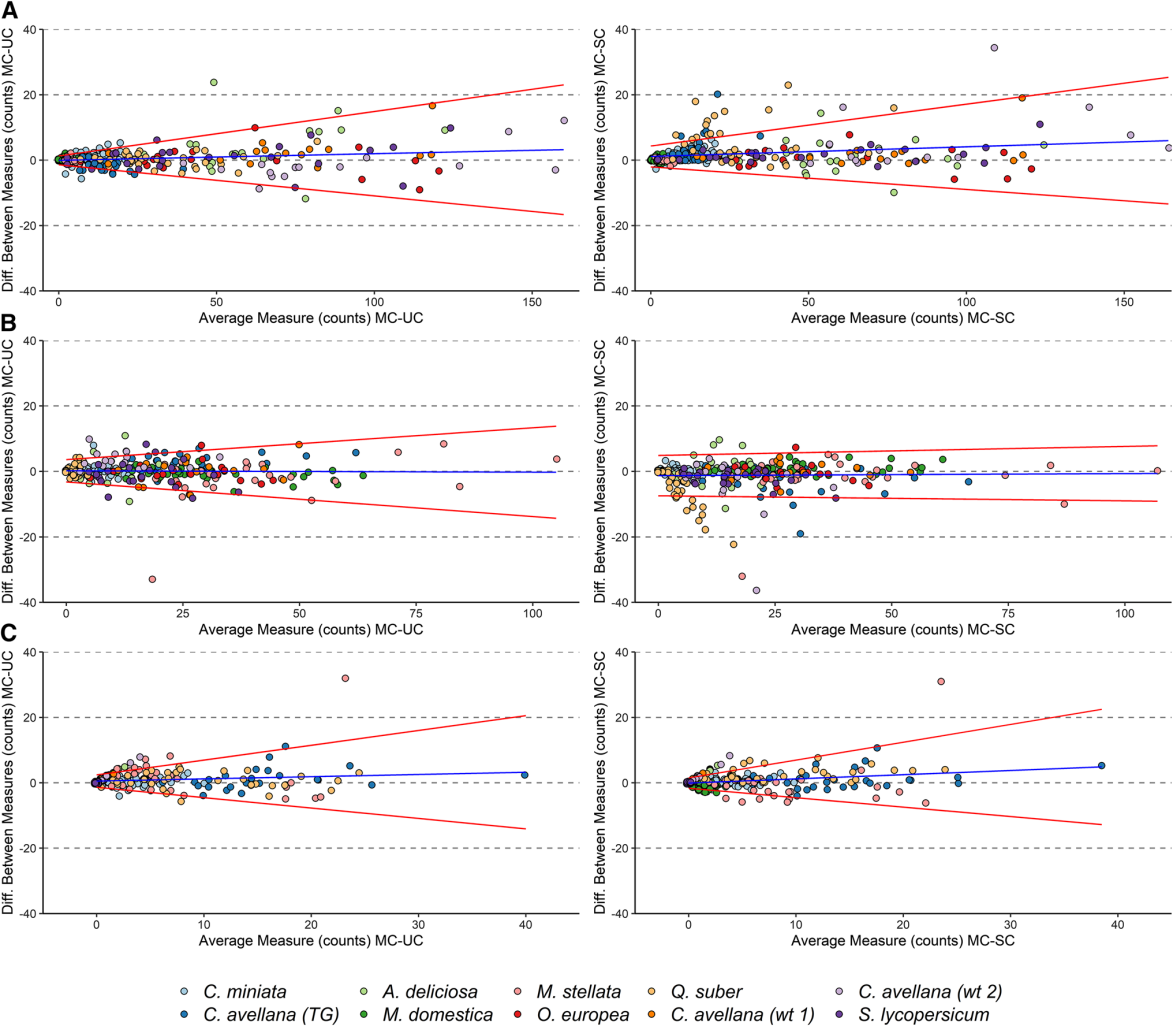
Bland–Altman plots for assessing classification accuracy between manual counting (MC) and unsupervised clustering (UC), on the left, and manual counting (MC) and supervised classification (SC), on the right. Viable **a**, dead **b** and sterile **c** pollen grains counted for each image were analysed separately. Colours correspond to plant taxa

Figure 6 shows the agreement between automated (UC and SC) and manual (MC) classification methods evaluated through the Bland–Altman approach for viable, dead and sterile pollen grains. The analysis of proportional bias detected a slight but significant tendency to underestimate viable and sterile pollen grains at higher average counts both for UC (respectively, *y* = 0.02 ×  − 0.07, *P* ≤ 0.001 and *y* = 0.07 ×  + 0.50, *P* ≤ 0.01) and SC (respectively, *y* = 0.03 ×  + 1.1, *P* ≤ 0.001 and *y* = 0.13 ×  − 0.09, *P* ≤ 0.0001). The error of automated classification was proportional to average counts, as heteroscedasticity was present for all the classes except for dead pollen in SC.

#### Pollen viability and sterility in the analysed plant taxa

**Fig. 7:**
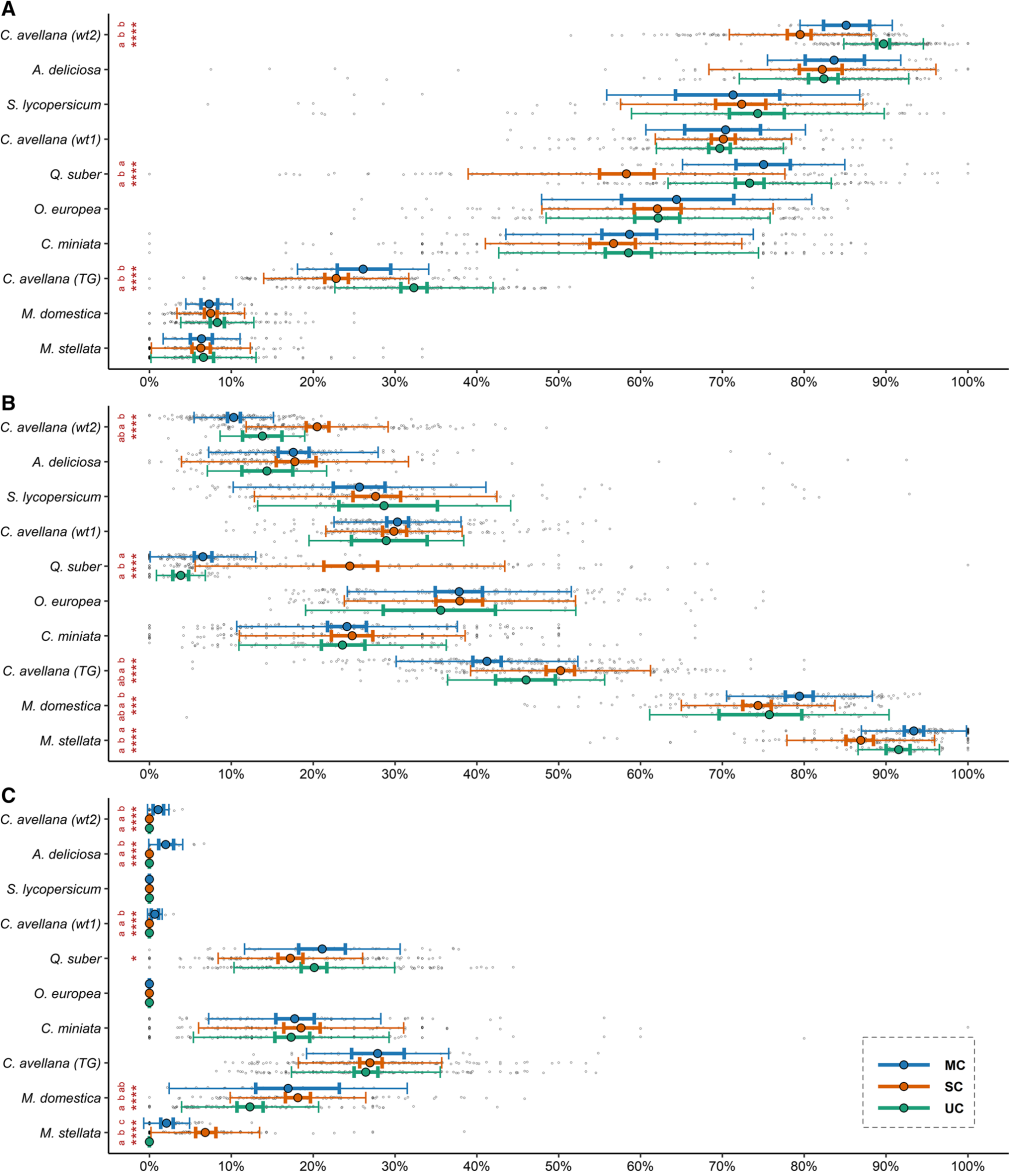
For each taxon, comparison of the percentages of viable **a**, dead **b** and sterile **c** pollen computed by the manual counting (MC), Fiji macro and unsupervised clustering (UC), CellProfiler pipeline and supervised classification (SC). Coloured dots correspond to average values, the error bars in bold display the bootstrapped confidence limits of the mean (95%), while the regular error bars display two standard deviations above and below mean values. Stars denote overall differences assessed using nonparametric Kruskal–Wallis test (**** *P* ≤ 0.0001, *** *P* ≤ 0.001, * *P* ≤ 0.05). Different letters mark significant pairwise differences based on the post hoc Wilcoxon signed-rank test (*P* ≤ 0.05). The grey scatterplot displays actual values

The labelling procedure allowed the differentiation between viable and dead pollen in all the considered taxa (Fig. 1), whereas sterile pollen was detected in *C. miniata*, *M. stellata*, *Q. suber*, *M. domestica*, *C. avellana* and *A. deliciosa*. Apart from the difference in labelling, the loss of viability was also accompanied by a decrease in overall pollen dimensions (Fig. 5). Smaller and collapsed grains, labelled differently depending on the species, were characteristic of plant taxa affected by male sterility. Pollen viability and sterility greatly varied among the analysed plant taxa (Fig. 7). When comparing cross-method average values, good viability levels were found for *C. miniata* (58%), *O. europea* (62%), *Q. suber* (67%), *S. lycopersicum* (73%), *C. avellana* (*wt1*) (70%), *A. deliciosa* (82%) and *C. avellana* (*wt2*) (85%). On the contrary, *M. domestica* (77%) and *M. stellata* (90%) showed very high percentages of dead pollen. High sterility levels affected *M. domestica* (15%), *C. miniata* (17%), *Q. suber* (19%) and *C. avellana* (TG) (27%). An important difference was detected in hazelnut, where the cultivar Tonda di Giffoni showed a higher pollen sterility compared to the wild types.

Looking at the departures from the manual procedure, viable pollen percentages measured with UC were significantly higher (*P* ≤ 0.05) in hazelnut TG and *wt2*. In addition, the SC method did not correctly differentiate among viable and dead pollen in *Q. suber* (*P* ≤ 0.05) (Fig. 7a, b).

The percentages of sterility were correctly detected by the two automated methods in hazelnut TG and *C. miniata*. In *Q. suber,* overall significant differences were found (*P* ≤ 0.05) but the pairwise comparison did not confirm the results (*P* ≥ 0.05). The biggest divergence was detected in *M. stellata*, where both UC and SC measured different amounts of sterile grains in comparison with the manual method (*P* ≤ 0.05) and in *M. domestica*, where sterility was underestimated by the UC method (*P* ≤ 0.05). Overall, as shown for wild type hazelnuts and *A. deliciosa*, both automated classification methods were not able to detect sterile pollen when present at low levels (Fig. 7c).

#### The FDA/PI pollen image dataset

**Fig. 8:**
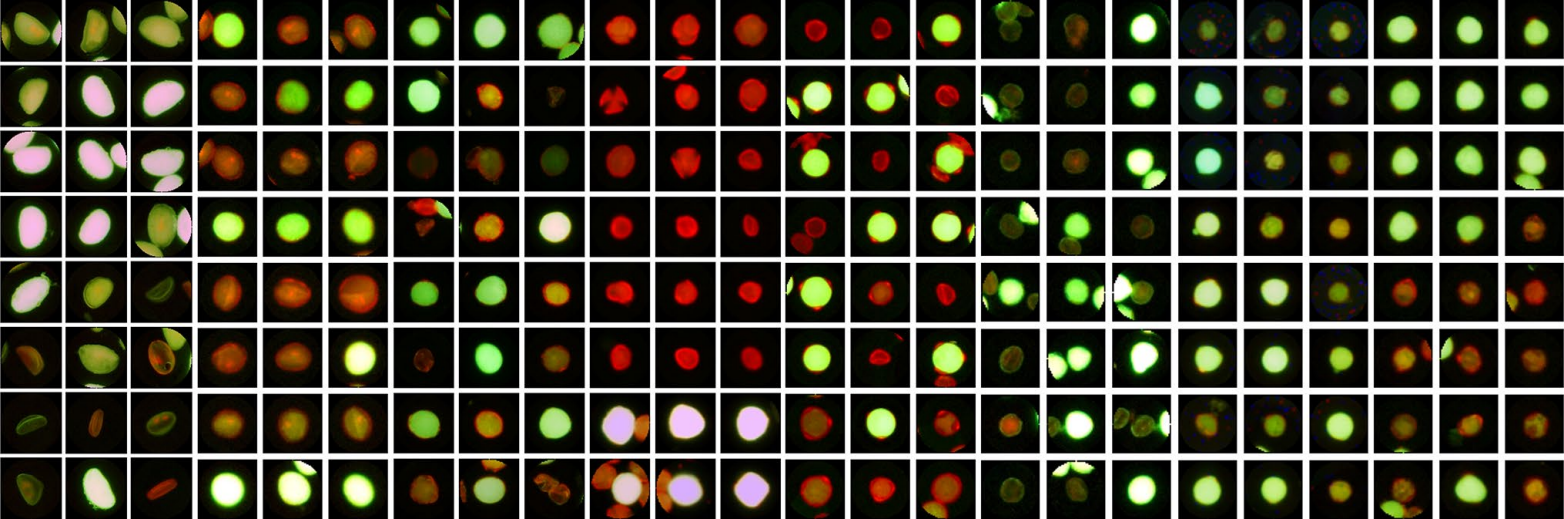
Examples of unscaled images of the FDA/PI pollen dataset

A total of 62,577 individual images were generated for pollen grains from eight plant species: *Corylus avellana* (19,563, 51 × 51 px), *Actinidia deliciosa* (10,312, 41 × 41 px), *Quercus suber* (8372, 59 × 59 px), *Solanum lycopersicum* (7097, 41 × 41 px), *Olea europea* (6567, 41 × 41 px), *Malus* domestica (5504, 61 × 61 px), *Magnolia stellata* (3055, 61 × 61 px), *Clivia miniata* (2107, 81 × 81 px). Each image was tagged with the plant species name. Examples of the generated pollen images are shown in Fig. 8. The complete image dataset can be accessed at 10.6084/m9.figshare.12758750.

## Discussion

The combination of FDA and PI dyes was able to label differently viable, dead and sterile pollen in all the analysed pollen taxa confirming the results obtained in other studies (Dupl’Áková et al. 2016; Greissl 1989; Regan and Moffatt 1990; Singh et al. 2015). Classification procedures ensured a thorough exploration of data and identified correctly viable, dead and sterile pollen. Automated methods increased significantly the statistical power of the pollen viability assay. Finally, a pollen image dataset was built to contribute to future research in computer vision applied to plant biology and reproduction.

### Pollen quality evaluation via FDA and PI labelling

Labelling results (Figs. 1 and 8) demonstrated that a general labelling procedure can be applied to different pollen taxa, though it has been suggested that specific media could improve FCR results in some plants (Nepi et al. 2010). Keeping samples at room temperature and in the dark sufficiently prevented colour fading for the time needed to acquire the images. An overall beneficial effect of pollen pre-hydration was also noticed during experiments, as non-pre-hydrated samples after thawing were generally unable to retain viability in liquid medium (data not shown). While pre-hydration treatment is advisable for stored pollen in order to prevent leakage of internal solutes in the medium and cell death, it can also be recommended for fresh samples as pollen viability is often correlated to pollen water content (Chichiriccò 2000; Shivanna 2003; Fonseca and Westgate 2005; Nepi et al. 2010). Nevertheless, some pollen types can be irreversibly damaged by excessive dehydration treatments and do not recover viability even after rehydration (Shivanna and Heslop-Harrison 1981; Lansac et al. 1994). The labelling technique was also effective at identifying anomalous and smaller pollen grains that were apparently void of the cytoplasm in some plant species (Figs. 1 and 8). This is particularly relevant as sterile pollen could be misclassified as generic dead pollen by researchers. Instead, they are results of different biological processes acting either during pollen development, yielding malformed pollen, or during and after pollen dehiscence, affecting the viability of pollen with a regular morphology.

Some hazelnut cultivars, including TG, are affected by reciprocal chromosome translocations, a condition that leads to the production of abundant sterile pollen (Salesses and Bonnet 1988; Marinoni et al. 2018). As expected, a considerable amount of shrunken pollen was present in hazelnut TG cultivar, whereas wild type pollen was well-developed and viable for the most part, suggesting the presence of a regular genetic background (Fig. 7). In our investigation, pollen sterility also characterized *C. miniata* and *M. domestica* (Fig. 7). Different levels of pollen sterility also characterized *M. domestica* “McIntosh” diploids and mixoploids and *C. miniata* subjected to drought and flood conditions (Yamburov et al. 2014; Podwyszyńska et al. 2016). Unfortunately, the genotype of the old apple tree that is conserved at the botanical garden of Turin could not be traced.

### Automation of the FDA/PI assay

One of the main issues in FDA-based assays is the high background fluorescence caused by the leakage of fluorescein from dead pollen grains, especially in conditions of low overall viability (Shivanna 2003). This phenomenon reduces the assay sensitivity and increases uncertainties in counting made either by humans (Aronne et al. 2001) or by computers (Novara et al. 2017). The image analysis workflows implemented here effectively reduced unwanted fluorescence through image pre-processing operations for illumination correction and background subtraction (Figs. 1 and 8). CellProfiler in particular showed higher versatility by the inclusion of multiple modules to handle these kinds of issues. In the comparison between manual and automated methods, the presence of proportional bias can be interpreted as an increasing tendency to underestimate total counts at higher pollen loads. Besides, heteroscedasticity means that method uncertainty is more pronounced when pollen count increases. These drawbacks were notable in both automated methods (Figs. 4 and 6). Some uncertainty can be expected even when counts are made by eye especially when objects get crowded on images (Aronne et al. 2001). On the other hand, the trend towards underestimation can be explained by the difficulty at identifying pollen within clumps that was handled by removing all the objects above a certain dimensional threshold in both Fiji macro and CellProfiler pipeline. Other authors also recorded a similar drop in performance of automated counting methods associated with clumped pollen (Go et al. 2019; Mudd and Arathi 2012). To reduce proportional bias, Tello et al. (2018) also implemented an approach based on dimensional and shape characteristics of identified grains. Separating overlapping objects is a complicated task, but capabilities in computer vision are steadily improving and possible solutions are starting to appear in the literature (Gallardo-Caballero et al. 2019; Cohen et al. 2017; Molnar et al. 2016).

Previous efforts on the automation of pollen viability assays identified different pollen conditions at the image analysis level. Tello et al. (2018) cleverly built a solution based on the separate counting of total and well-developed pollen on the red and green channels, respectively. Sterile grains were found by simple subtraction. This was allowed because Alexander’s method stains sterile and well-developed pollen strictly in a selective way, with no cross-channel colour contamination (Alexander 1969). A separate counting of aborted and well-developed pollen on images was also built for aniline blue staining (Mudd and Arathi 2012).

Following another approach, we classified data obtained through the analysis of FDA/PI fluorescent images using supervised and unsupervised methods. A similar classification performance of MC and UC was recorded for viable, dead and sterile pollen (Fig. 6). Moreover, by analysing whole image samples, automated methods generally improved confidence concerning average values of sterile, dead and viable pollen (Fig. 7).

CellProfiler Analyst (Dao et al. 2016) simplified the process of data exploration and supervised classification through an easy-to-use graphical interface (Fig. S1). Supervised classification requires the a priori knowledge of relevant phenotypes among the detected objects (Smith et al. 2018). Therefore, if no previous knowledge on the sample is available, under-represented populations are likely to be ignored. This was the case in our study where supervised classification was unable to detect sterile pollen when present in very small quantities (Fig. 7). To address this problem, other classification tools that implement data mining approaches for the discovery of rare populations could be used (Smith et al. 2018). The number of objects chosen as baseline for the training process was enough to guarantee an overall acceptable level of classification accuracy (Fig. S1 C). Increasing the training effort would easily help in significantly reducing the recorded uncertainties.

Clustering is a method for data exploration that does not require previous annotation and that can help in looking for relevant populations. An unsupervised method for the determination of pollen viability was recorded just once in the literature, where rice pollen was classified through k-means clustering with a fixed number of clusters (Go et al. 2019). We implemented a feature extraction procedure to simplify the automatic definition of the best number of clusters and to apply a fully automated hierarchical clustering based on Manhattan distances (File S3). This procedure allowed not only to explore data but also to automatically find relevant classes for most of the considered taxa (Fig. S2). Integrated density, area and skewness were crucial for the separation of viable, dead and sterile pollen populations (Fig. 5). Similar to the results of the supervised method, unsupervised clustering was unable to detect very low amounts of sterile pollen grains (Fig. 7). Clustering algorithms are usually optimized to identify either main clusters or rare cases; therefore, the sequential application of multiple algorithms might be beneficial (Weber and Robinson 2016).

Fluorescence variation among single images or image batches due to heterogeneous experimental conditions can introduce classification errors in supervised and unsupervised techniques. Automated image acquisition systems can contribute to improving reproducibility and lowering the amount of time required for the analysis. In addition, normalization techniques can be applied to images and to object features to reduce signal unevenness and classification errors (Kothari et al. 2014). The Fiji macro implemented a basic image normalization method through histogram equalization (Štruc and Pavešić 2017) for enhancing pollen recognition and clustering reproducibility. Future works could try to apply algorithms for feature normalization (Kothari et al. 2014) in order to further refine classification performance.

As a final recommendation, for the overall better performance, the ease of use and good versatility which were shown in this study, it could be supported the adoption of CellProfiler/CellProfiler Analyst (or other classification tools) as the software of choice for the automated evaluation of pollen viability employing the FDA/PI labelling. The training set for the supervised classification process should be at least made of 40 objects for each class. Random forest usually offers a good balance between computing time and classification accuracy, nevertheless other models should be considered and tested for specific needs (Kuhn 2008; Gallardo-Caballero et al. 2019).

### The image dataset

The availability of a large number of labelled images is a prerequisite for the employment of advanced image analysis methods based on deep learning, which are able to discover object features from images and classify them in an integrated and highly effective approach (Tsaftaris et al. 2016; Zhao et al. 2019). With this study, we also provide the first image dataset on pollen quality in fluorescent microscopy. With 62,577 images, it is the largest palynological database ever built, albeit limited to eight plant species. Available image databases contain pollen grains imaged in bright-field microscopy and do not exceed 13,500 entities (Duller et al. 1997; Ranzato et al. 2007; Gonçalves et al. 2016; Battiato et al. 2020). Future work will concentrate on the annotation of the viability class (viable, dead and sterile pollen) in order to provide a fully annotated dataset for the identification and classification of pollen grains labelled with FDA and PI.

## Conclusions

The proposed methods and the publicly available dataset open new opportunities in pollen viability evaluation, both quantitatively and qualitatively, and provide a foundation for an automated and low-cost tool to deepen knowledge of pollen biology and ecology with promising applications in agriculture and plant breeding.

## Electronic supplementary material

Below is the link to the electronic supplementary material.

Supplementary file1 (CPPROJ 98 kb)

Supplementary file2 (IJM 3 kb)

Supplementary file3 (R 14 kb)

Supplementary file4 (DOCX 40416 kb)

Supplementary file5 (DOCX 16 kb)
