# Supplementary material for: Quantitative methods in microscopy to assess pollen viability in different plant taxa
Source: Plant Reprod. 2020 Oct 29;33(3-4):205–19. doi: 10.1007/s00497-020-00398-6 (PMC7648740; doi:10.1007/s00497-020-00398-6)
Supplement: Supplementary file 4 — Supplementary file4 (DOCX 40416 kb) [file 497_2020_398_MOESM4_ESM.docx]

**
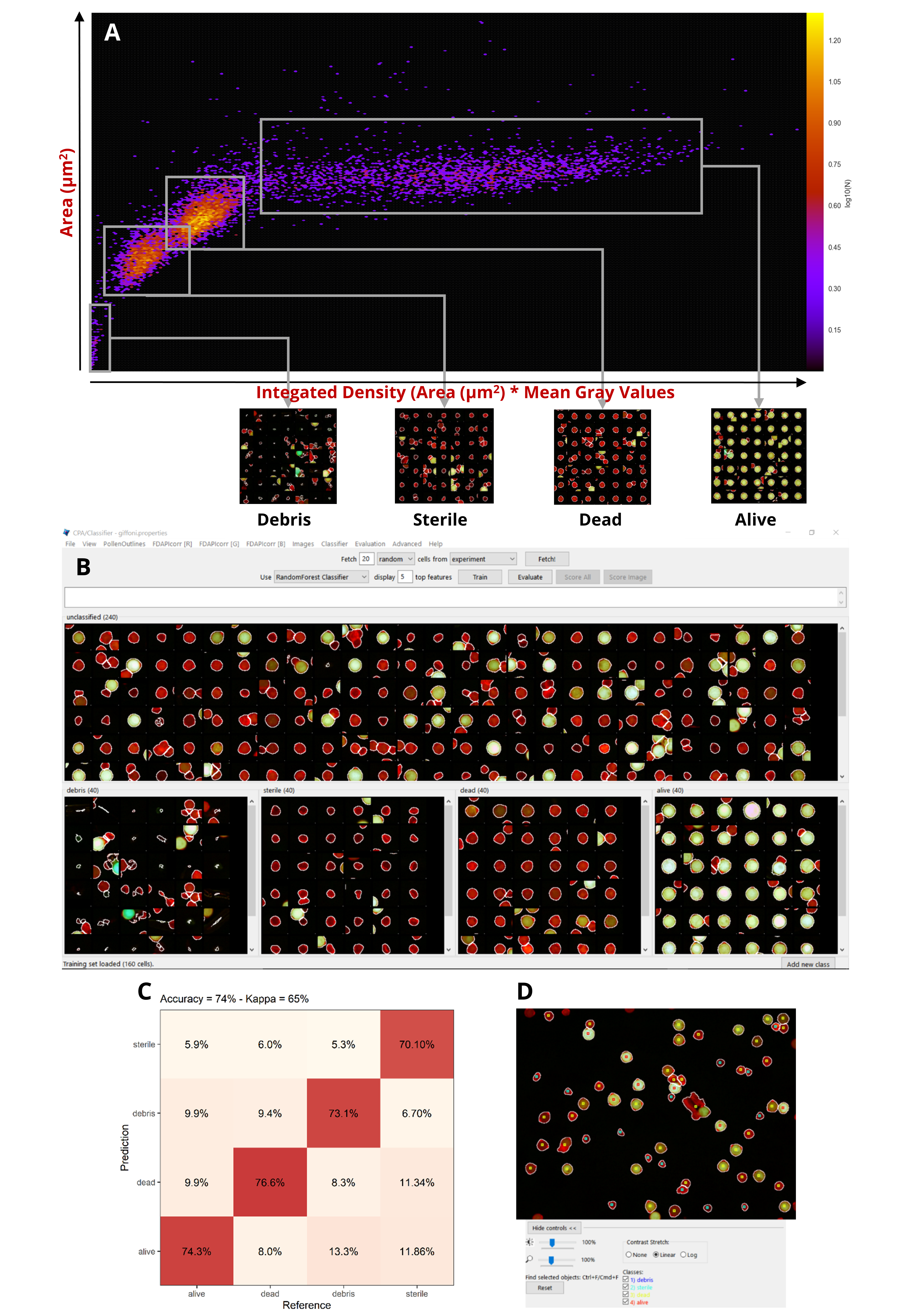
**

Fig. S1.Example of the analysis performed using CellProfiler Analyst software for the supervised classification of pollen viabilty in *C. avellana* (TG). (**A**) Data exploration and manual gating of relevant populations. (**B**) Definition of viability classes (viable, dead, sterile and debris) (**C**) Evaluation of the random forest overall classification performance (**D**) Viability detection on a sample image after automated classification.





Fig. S2.The populations of viable, dead and sterile pollen grains identified for each species by the unsupervised clustering of pollen features measured by the Fiji macro.
