# Supplementary material for: Quantitative methods in microscopy to assess pollen viability in different plant taxa
Source: Plant Reprod. 2020 Oct 29;33(3-4):205–19. doi: 10.1007/s00497-020-00398-6 (PMC7648740; doi:10.1007/s00497-020-00398-6)
Supplement: Supplementary file 5 — Supplementary file5 (DOCX 16 kb) [file 497_2020_398_MOESM5_ESM.docx]

Table S1. Per taxon total number of counted pollen grains and analysed images (in brackets) for the manual procedure (MC), CellProfiler and Fiji software. In the last two columns, only the images used for manual counting were considered for the comparison and in brackets the shift from the manual counting was computed.

| Taxon | MC (n° of images) | CP (n° of images) | Fiji (n° of images) | CP (-MC) | Fiji (-MC) |
| --- | --- | --- | --- | --- | --- |
| *Clivia miniata* | 1564 (76) | 2011 (120) | 2062 (120) | 1441 (-123) | 1467 (-97) |
| *Corylus avellana* (TG) | 1528 (27) | 7757 (151) | 7621 (151) | 1482 (-46) | 1462 (-66) |
| *Corylus avellana* (wt1) | 1531 (15) | 13251 (123) | 13074 (123) | 1444 (-87) | 1479 (-52) |
| *Corylus avellana* (wt2) | 1574 (15) | 17531 (150) | 17478 (150) | 1521 (-53) | 1518 (-56) |
| *Actinidia deliciosa* | 1502 (19) | 9856 (119) | 9477 (119) | 1596 (94) | 1396 (-106) |
| *Magnolia stellata* | 1519 (46) | 2759 (108) | 2701 (108) | 1530 (11) | 1504 (-15) |
| *Malus domestica* | 1539 (31) | 4724 (107) | 4552 (107) | 1404 (-135) | 1534 (-5) |
| *Olea europea* | 1527 (19) | 6642 (90) | 6601 (90) | 1489 (-38) | 1542 (15) |
| *Quercus suber* | 1510 (34) | 4422 (128) | 4615 (128) | 1524 (14) | 1469 (-41) |
| *Solanum lycopersicum* | 1546 (23) | 7167 (91) | 7148 (91) | 1551 (5) | 1549 (3) |
| Total | 15340 (305) | 76120 (1187) | 75329 (1187) | 14982 (-358) | 14920 (-420) |
